# Idiographic analyses of motivation and related processes in participants with schizophrenia following a therapeutic intervention for negative symptoms

**DOI:** 10.1186/s12888-020-02824-5

**Published:** 2020-09-25

**Authors:** Bénédicte Thonon, Evelyne Van Aubel, Ginette Lafit, Clara Della Libera, Frank Larøi

**Affiliations:** 1 Faculty of Psychology, Speech and Laguage Therapy and Education Sciences, Psychology and Neuroscience of Cognition Research Unit, University of Liège, Place des Orateurs 1, 4000 Liège, Belgium; 2 Department of Neurosciences, Center for Contextual Psychiatry (CCP), Katholieke Universiteit Leuven, Kapucijnenvoer 7, 3000 Leuven, Belgium; 3 Department of Psychology, Research Group of Quantitative Psychology and Individual, Differences, Katholieke Universiteit Leuven, Kapucijnenvoer 7, 3000 Leuven, Belgium; 4 Department of Biological and Medical Psychology, University of Bergen, Jonas Lies vei 91, 5009 Bergen, Norway; 5 NORMENT – Norwegian Center of Excellence for Mental Disorders Research, University of Oslo, Kirkeveien 166, 0450 Oslo, Norway

**Keywords:** Apathy, Intervention, Ecological momentary assessment, Timeseries analyses, dynamics, Single case

## Abstract

**Background:**

Motivational negative symptoms hinder quality of life and daily functioning of individuals with schizophrenia spectrum disorders. A recently developed intervention, Switch, has shown promising effects on negative symptoms and functional outcomes. Switch targets multiple cognitive, emotional and behavioural processes associated with motivation and goal directed behaviours. We aimed to investigate its effects on motivation and associated processes in a naturalistic setting, and to explore the dynamics between the processes.

**Methods:**

We used a single case approach (*n =* 3), with a pre-post and follow-up assessment design, which also included ambulatory assessments (experience sampling method, ESM; and step count). We computed autoregressive lag 1 models to evaluate the effects of the intervention on daily motivation levels and related processes, descriptive pie-charts, and vector autoregressive modelling to reveal the dynamics of the processes over time.

**Results:**

The intervention was beneficial for each participant according to traditional evaluations of motivational negative symptoms, apathy, daily functioning and quality of life. The effects on the ESM variables revealed distinct outcomes for each individual. The dynamics between the various processes differed between participants, and fluctuated within participants (when comparing baseline, intervention phase, and follow-up).

**Conclusions:**

This study used an innovative approach to look at the effectiveness of an intervention. The intervention seems to lead to meaningful improvements in motivational negative symptoms and functional outcomes. The mechanisms of change need to be further investigated.

**Trial registration number:**

ClinicalTrials.gov, NCT04325100. Registered 27 March 27, 2020 -retrospectively registered.

**Reporting:**

Guidelines from the Transparent Reporting of Evaluations with Non-randomized Designs (TREND) statement were followed.

## Background

Negative symptoms are highly prevalent in individuals with schizophrenia [1]. These symptoms are generally understood as comprising two factors: expressive deficits, including blunted affect and alogia; and motivational or experiential deficits, including anhedonia, avolition, and asociality [2, 3]. Motivational negative symptoms appear to be the main obstacle to daily functioning [4, 5] and quality of life [6]. To date, psychological interventions as well as pharmacological treatments have shown limited or inconsistent effects on negative symptoms [7, 8]. One way of remedying this is to better understand the underlying processes of motivation in schizophrenia in order to provide tailor-made interventions that focus on those particular processes.

**Fig. 1:**
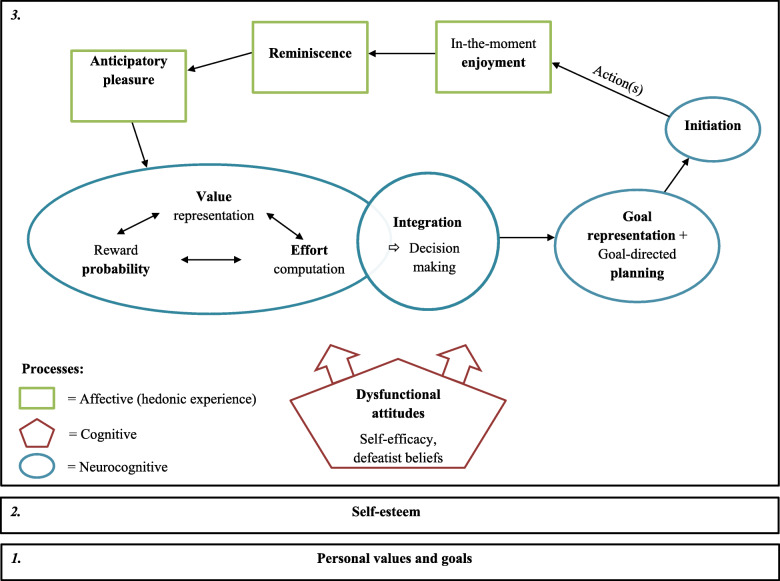
Three-level model of motivation (reproduced with permission from [9])

In a previous study [9], we presented a model of motivation in schizophrenia that integrates various emotional/hedonic, (neuro) cognitive and behavioural processes that are related to motivation and goal-directed behaviours and that are often dysfunctional in individuals with schizophrenia. This model was predominantly inspired by the model developed by Kring and Barch, which follows the course of hedonic processes, from the anticipation of a reward to its obtainment [10]. The model we describe here integrates additional processes (e.g., dysfunctional attitudes) and furthermore is multilevel. Figure 1 presents this three-level model. The first two levels are at the foundation of the model and include personal values and goals on the first level, and self-esteem on the second level. The third level narrows in on a chosen value or goal, and targets those processes that underpin motivation. It starts with the anticipation of pleasure, which is mainly sensory. The next step is largely cognitive and requires an estimation of the effort, the value and the probability of attainment of the chosen goal. These processes can be influenced by dysfunctional attitudes (e.g., defeatist beliefs or low self-efficacy) and thus potentially altering the decision to take action. Once the motivated decision has been taken, planning skills come into play, as well as action initiation abilities. In-the-moment enjoyment occurs while advancing towards or reaching the chosen goal. Finally, the experience of successfully moving towards one’s goal can be recalled (reminiscence) and can feed further anticipation (for a more detailed description and an illustration, see [9]).

We developed an intervention, Switch, that targets the multiple elements and processes identified in the aforementioned model [9], using various strategies that have proven to be beneficial as delivered separately or in other clinical populations (e.g., [11–16]). A pilot study was conducted with 8 participants with schizophrenia spectrum disorders who followed Switch for a year (30 sessions on average). We found significant moderate to large positive effects on motivational negative symptoms and functional outcomes. The novelty of the Switch intervention is that it is specifically designed to address motivational negative symptoms and therefore the multiple and various processes related to these symptoms. The strength of Switch is also its recovery approach [17], with a focus on autonomy and personal resources, goals, and values.

In the current study, we wished to further validate Switch, however, using a different set-up by providing the intervention twice a week for 2 months, in order to meet certain time constraints of psychiatric health care. Additionally, we aimed to better understand the dynamics of processes related to motivation across time. In order to do this, we used a single case design which allows a thorough investigation of mechanisms of change by applying repeated measurements of the processes of interest [18]. The high internal validity of this approach can even further improve by including intensive repeated assessment, such as the Experience Sampling Method (ESM [19, 20];) or other ambulatory assessment strategies that imply continuous and objective measurement of activity [21, 22]. Such intensive repeated assessment can help understand the dynamic interconnections between the variables of interest over time. Furthermore, ESM diminishes the risk of retrospective recall biases, and allows a more natural and nuanced evaluation of emotions, cognitions and behaviours in everyday life and in the person’s real environment (vs. laboratory and clinical settings) [22].

The first aim of this study was to evaluate the effectiveness of a shorter version of the Switch intervention in individuals with schizophrenia and schizoaffective disorder provided in a naturalistic setting. We expected (1) an improvement on motivation/apathy, quality of life and functional outcomes (primary outcomes), after the intervention and/or at follow-up; (2) an improvement on the various cognitive, emotional and behavioural processes identified in our model and targeted in the intervention (ESM variables), during the intervention and/or at follow-up; (3) an increase in step count as this has been shown to be an objective proxy measure of negative symptoms [23], during the intervention and/or at follow-up. Second, we aimed to explore the dynamics of the processes associated with motivation (ESM variables) before the intervention, during the intervention and at follow-up.

## Methods

### Participants

Participants were recruited in March 2019, via referral from a mental health community centre in the French speaking community of Belgium, where the recruitment, assessments and intervention took place. Inclusion criteria for the present study were: aged between 18 and 65, met DSM-5 criteria for schizophrenia or schizoaffective disorder [24] and a good understanding of French. Exclusion criteria were: presented an unstable clinical picture (i.e., no acute positive symptoms); evidence of a significant change in medication within 1 month prior to baseline assessment; history of severe brain trauma or epilepsy; comorbid intellectual disability; and moderate or severe substance use disorder other than tobacco (according to the DSM-5; i.e., showing 4 or more symptoms). The head psychiatrist from the mental health community centre was familiar with the inclusion and exclusion criteria of the study and other relevant details (e.g., the need for participants to provide informed consent). Thereafter, out of a pool of 60 patients, six candidates who fulfilled the criteria were contacted, introduced to the study and asked if they accepted to be contacted by the main investigator. Three accepted. Next, the main investigator contacted these three candidates by phone and presented the study. The participants were then seen in person and received a thorough explanation of the evaluation protocol, the intervention and their rights as participants in the study. They were invited to read the information sheet (including repetitive disclosure and emphasis of key points, as recommended by [25]), ask any questions that they might have, and sign the informed consent if they accepted to participate. Three participants enrolled in the study and were assigned to the Switch intervention in April 2019. One participant was lost to follow-up in October 2019.

The study was approved by the Liege University Hospital Ethics Committee (B707201629105). Sociodemographic and clinical characteristics of the participants are reported in Table 1.

**Table 1:** *Sociodemographic characteristics*

Individual	2i-1	2i-2	2i-4
Age	29	34	39
Gender	Female	Male	Female
Diagnosis	SZ	SZ	SZ
Illness duration (years)	10	10	7
Education (year)	10	12	14
Living Conditions	With partner	Supervised housing	With family
Work	/	/	/
Switch (number of sessions + booster session)	12 + 1	15 + 1	10 + 0
Medication / dose / CPZeq	Aripiprazole / 2.5 mg/day / 50 mg	Aripiprazole / 200 mg/month / 142.86 mg	Olanzapine / 350 mg/3 months / 277.47 mg

*CPZeq* Chlorpromazine equivalents (mg/day) [26]

### Study design and procedure

**Fig. 2:**
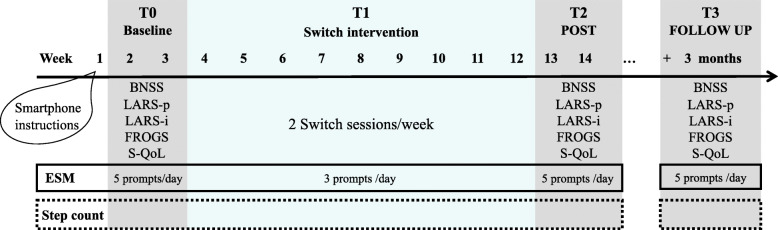
Design of the study and assessment procedure. BNSS = Brief Negative Symptom Scale; LARS = Lille Apathy Rating Scale patient and informant; FROGS = Functional Remission of General Schizophrenia, S-QoL = Schizophrenia Quality of Life questionnaire

Participants underwent three types of evaluation: traditional assessment scales of motivational deficits, apathy, quality of life and daily functioning; ambulatory assessment including ESM (i.e., questionnaires); actigraphy (step count). Participants were evaluated on the traditional assessment scales before the intervention (Pre), after the 2-months intervention (Post), and 3-months after the end of the intervention (Follow-up). ESM and actigraphy were used before (T0), during (T1), and after the intervention (T2), as well as at follow-up (T3). Figure 2 provides a visualisation of the study design and procedure. Participants received feedback on all evaluations at the end of the study.

### Traditional evaluation (primary outcomes)

Assessments were completed by trained evaluators. Participants were told to refer to the last 2 weeks when completing the following scales:

*Brief Negative Symptoms Scale (BNSS)* [27]. The French version of the BNSS was used in this study [28]. The BNSS assesses expressive and motivational negative symptoms. Only the BNSS – Motivation subscale was used, which is the mean of the following subscales: anhedonia (intensity of pleasure during activities, frequency of pleasure, intensity of expected pleasure from future activities), asociality (behaviour, internal experience), avolition (behaviour, internal experience). Each item is scored from 0 to 6 (0 = no impairment; 1 = very slight; 2 = mild; 3 = moderate; 4 = moderately severe; 5 = marked; 6 = severe). A blinding procedure was used: participants’ interviews were recorded and each video or sound recording was evaluated at the end of the study by two independent evaluators who were unaware of the recordings’ assessment time. The BNSS possesses excellent internal, convergent and discriminant validity [28], excellent test-retest and interrater reliability [27], and good sensitivity to change [29].

*Lille Apathy Rating Scale – Patient version (LARS-p)* [30]. The LARS is a semi-structured interview that evaluates the different dimensions (cognitive, emotional and behavioural) of apathy through the following subscales: everyday productivity, interests, taking initiatives, novelty seeking, voluntary actions, emotional responses, concern, social life and self-awareness. The total score ranges from − 36 to 36 ([− 36; − 22] = absence of apathy; [− 21; − 17] = tendency towards apathy; [− 16; − 10] = moderate apathy; [− 9; 36] = severe apathy). The LARS-p possesses a high level of inter-rater reliability and satisfactory internal consistency [31].

*Schizophrenia - Quality of Life questionnaire (S-QoL)* [32]. The S-QoL is a 41-item questionnaire that evaluates life satisfaction regarding psychological wellbeing, self-esteem, family relationships, relationships with friends, resilience, physical wellbeing, autonomy and sentimental life. Items are rated on a 5-point Likert scale (1 = much less satisfied than expected; 2 = less satisfied; 3 = slightly less satisfied; 4 = as satisfied; 5 = more satisfied). The total score ranges from 0 to 100, higher scores indicating better subjective quality of life. The S-QoL shows good internal and convergent validity, excellent test-retest reliability [32], and good sensitivity to change [33].

Informants were interviewed to provide an external understanding regarding participants functioning. The informant for participant 2i-1 was her husband; the informant for participant 2i-2 was the head of his supervised housing; participant 2i-4 did not wish to involve an informant. Informants completed the following two scales:

*Lille Apathy Rating Scale – Informant version (LARS-i)* [34] (see the patient version for a description). The LARS-i shows high internal consistency and concurrent validity, as well as high levels of test-retest and inter-rater reliability [34].

*Functional Remission of General Schizophrenia (FROGS)* [35]. The FROGS is a measure of daily life outcomes, which evaluates level of functioning in 5 different domains: daily life, activities, relationships, quality of adaptation, and health and treatment. Via a semi-structured interview with the informant, each item is assessed on a 5-point scale: 1 = does not do; 2 = does partially; 3 = does a significant part; 4 = does almost all of it; 5 = does perfectly. The total score ranges from 19 to 95. The threshold score for remission is 61 [36]. The FROGS possesses high concurrent validity and internal consistency [35].

### Ambulatory assessment (secondary outcomes)

#### ESM questionnaire

Prior to the start of the study, the participants received extensive explanations regarding the ESM procedure. Participants installed the MetricWire app (https://metricwire.com/) and were logged in with a sham email address. Participants filled in an example-questionnaire with the investigator who explained all the questions and their possible answers.

For the 14 consecutive days of the baseline phase, participants were prompted by the app MetricWire five times a day at pseudo-random time points, within 3-h time frames between 7.30 a.m. and 10.30 p.m. Each prompt invited the participant to open the app and answer the questionnaire referring to what he/she was experiencing just before the prompt. The participants had 20 min to fill in the questionnaire and they received a reminder after 10 and 15 min. During the 2 months of intervention, in order to reduce the burden on the participants, the number of prompts was reduced to three per day, within 5-h time frames. After the end of the intervention, participants were prompted again 5 times per day for another 2 weeks. As participants did not complete enough questionnaires after the end of the intervention, the post-assessment ESM observations (T2) were not taken into consideration.

Table 2 presents the ESM questionnaire that was developed based on the different variables included in the motivation model described in the introduction. It was created following guidelines from Kimhy et al. [37]. The questionnaire included 14 questions, plus three optional branched questions, i.e., determined by the participant’s answer to a previous question.

**Table 2:** *ESM Questionnaire*

Variables	Questions	Rating
Mood	I feel …	1–7 Likert; 1 = Unhappy; 4 = Neutral; 7 = Happy
Discouraging beliefs	Discouraging thoughts are crossing my mind.	1–7 Likert; 1 = Not at all true; 7 = Totally true
*Coping (only if Q3 > 4).*	*How am I dealing with these thoughts?*	*I’m not. / I’m distancing myself from them. / I’m trying to use more constructive thoughts. / I’m looking for concrete solutions. / Other:*
*Coping (other)*	*You indicated “other”. What precisely do you do to deal with these discouraging thoughts?*	*Free text*
Confidence	I feel confident.	1–7 Likert; 1 = Not at all true; 7 = Totally true
Motivation	I feel motivated.	1–7 Likert; 1 = Not at all true; 7 = Totally true
Energy	I have energy.	1–7 Likert; 1 = Not at all true; 7 = Totally true
Social	Who am I with?	Stranger(s), other / Acquaintance(s) / Colleague(s) / Friend(s) / Family / Partner / I’m alone
Activity	What am I doing?	Nothing / Rest, passive activity (TV, internet, reading...) / Transport / Hygiene, household, grocery, meal / Social activity/interaction / Leisure / Physical activity / Work, study, training, attending a workshop / Other
*Activity (other)*	*Specify your activity:*	*Free text*
Initiation	Select the option that best corresponds with your situation:	I am the one who spontaneously started this activity. / Someone else encouraged me to start this activity. / I am not doing anything in particular.
Present enjoyment	I feel some pleasure in what I am doing.	1–7 Likert; 1 = Not at all true; 7 = Totally true
Wanting to give up	I feel like giving up this activity.	1–7 Likert; 1 = Not at all true; 7 = Totally true
Activity’s meaning (value)	This activity is important to me.	1–7 Likert; 1 = Not at all true; 7 = Totally true
Effort	This activity requires some effort.	1–7 Likert; 1 = Not at all true; 7 = Totally true
Reminiscence	Since the last prompt, I have been recalling pleasant past events.	1–7 Likert; 1 = Not at all, I have been thinking of *unpleasant* events; 4 = I have not been particularly thinking about the past; 7 = Absolutely, I have been thinking a lot about pleasant past events.
Projection into the future	Since the last prompt, I have been looking forward to some activities or events.	1–7 Likert; 1 = Not at all, I have been *apprehending* the future; 4 = I have not been particularly thinking about the future; 7 = Absolutely, I have been greatly looking forward to the future.

*Note:* Items have been translated from French into English.

*Italics:* conditional questions (branching).

The categorical ESM outcomes of interest were: activity’s meaning, motivation, mood, confidence, and savouring. Activity’s meaning, effort, energy, mood, and confidence represent each a single item from the ESM questionnaire. Motivation is a composite measure of the items motivation and wanting to give up (reverse coded). Savouring is a composite measure of present enjoyment, reminiscence, and projection into the future.

Nominal ESM outcomes of interest were coping strategies (in the presence of discouraging beliefs), social contact, activity and initiation. For further details and label descriptions of the ESM measures, please refer to Table 2.

#### Step count

Participants were provided with an activity band (MiBand 3, Xiaomi) which they had to wear at all times (day and night) during the different phases of the study (baseline, intervention, post-measurement, and follow-up). The band is waterproof and has a battery autonomy of approximately 20 days. A MiFit sham account was created in order to synchronize the activity band with the app on the participant’s smartphone. The MiFit app provided the total amount of steps per day.

### Intervention

Switch was delivered by the main investigator, a trained psychologist and psychotherapist. The individual sessions lasted 1 h and were given twice per week for 2 months, in the participants’ local mental health centre.

**Fig. 3:**
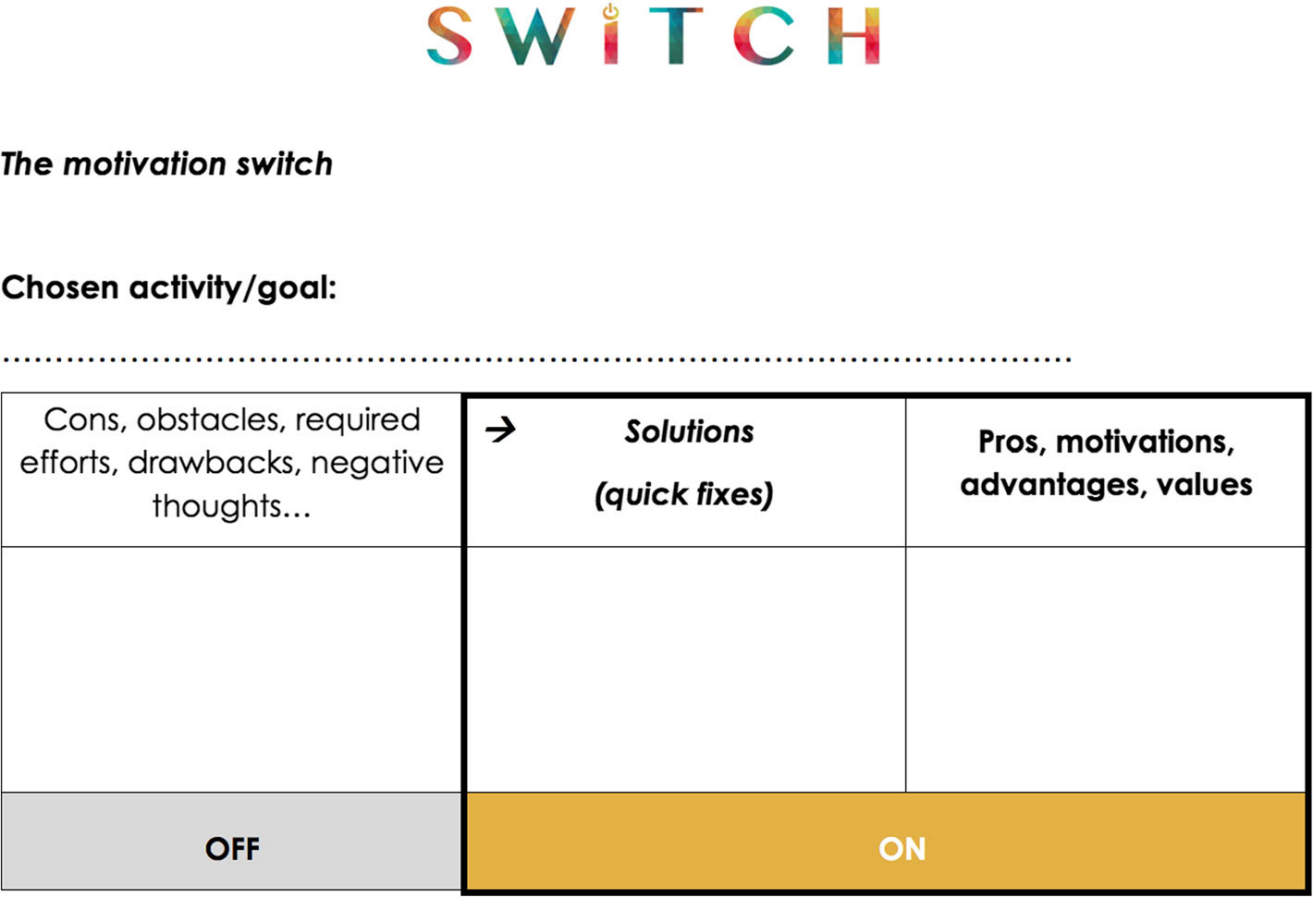
The motivation’s switch. Updated version of the decisional balance

The first sessions were dedicated to building a therapeutic alliance, getting to know the person and identifying personal resources, goals and values (i.e., addressing the first two levels of the motivation model, see 1. Introduction). Strategies were then taught in order to help the person to engage in behaviours directed towards these chosen goals and values (i.e., moving to the third level of the model). Multisensory “imagery” was used to help to look forward to the future (i.e., pleasure anticipation). This type of projection into future actions/goals included not only visualising the scene (i.e., the person her/himself, the context, the actions), but also imagining the possible sounds, physical sensations, smells, flavours, pleasant emotions, constructive thoughts, etc. The imagery thus goes through the different senses, in order to increase the possibility of experiencing pleasure and to help identify what modality generates the more pleasure – and consequently that has the higher motivational power. The imagery could focus on the process (e.g., baking a cake) or the result (e.g., eating the cake). A restructured decisional balance tool was used to address the effort-value computation. The “motivation’s switch”, as can be seen in Fig. 3, was used to identify all the reasons why the person would not engage in a certain activity (including potentially discouraging thoughts, required effort), and all the good reasons why she/he would engage in that activity. Additionally, a column was used to indicate quick solutions for the smaller obstacles that were identified. The solutions and pros represent the “ON” part of the switch, which is highlighted relative to the cons’ column, which represents the “OFF” part of the switch. The cons column potentially included obstacles and dysfunctional attitudes that needed further attention. Participants were then guided in solution-seeking strategies. Furthermore, significant discouraging thoughts and low self-efficacy were challenged using cognitive restructuring (e.g., generating more constructive thoughts) and/or a cognitive defusion approach (e.g., using metaphors, training mindfulness). Help in planning and initiation strategies (electronic reminders, implementation intention, post-its …) addressed the subsequent steps in the model. Finally, participants were invited to use various reminiscence strategies (e.g., sharing of experience with others, keeping a diary, looking at photos, buying souvenirs) to increase positive memories and boost motivation for new actions or goals to engage in.

Each participant learnt the different strategies in relation to their individual goals and needs. A folder which presented the rationale for each strategy was given to the participants. Take-home assignments were given and stored in the same folder. Participants were also given small cards (that could fit in their wallet) containing the key elements of each strategy. Furthermore, during the last 3 weeks of the intervention, the participants received daily triggers (via the MetricWire app) inviting them to look forward to coming events/activities (morning trigger) and to look back at their day and reminisce about positive incidents (evening trigger). The morning trigger included an mp3 that could be listened to from the app and that provided a guided multi-sensory projection into the future. Finally, a booster session took place around 45 days after the end of the intervention, consisting mainly of a reminder of the different strategies and a troubleshooting of possible obstacles.

The complete manual for the Switch intervention and the participant booklet (both in French) can be requested from the first author.

### Analyses

#### Aim 1: treatment effects of switch on motivation and related processes

We used effect size coefficients to report changes in BNSS, LARS-p, LARS-i, FROGS and S-QoL scores. We created effect size coefficients for the composite scores of motivation/apathy and of QoL/functioning. Motivation/apathy represented the mean of four scores from the BNSS-Motivation (i.e., two scores from the two blinded evaluators), the LARS-p and LARS-i. QoL/functioning included the two scores from the S-QoL and the FROGS. First, all variables were rescaled to fit a 7-point Likert-scale (0 to 6) in order to have comparable scores amongst the different scales and to compute effect sizes. The following equation was used to calculate the Cohen’s *d* statistics effect size coefficient: $\frac{\overline{X_0}-\overline{X_1}}{S_{pooled}}$, where *S*_pooled_ equals $\frac{\sqrt{\left({n}_0-1\right){\left({SD}_0\right)}^2+\left({n}_1-1\right){\left({SD}_1\right)}^2}}{n_0+{n}_1-2}$, where *n* represents the number of observations (i.e. 4 observations for Motivation/Apathy, and 2 observations for QoL/functioning) and *SD* the within-person standard deviation. This coefficient thus takes into account the number of observations and the standard deviation within each phase (pre and post, or pre and follow-up) and within each participant.

To further investigate the effects of the intervention, we examined whether Switch had an effect on the ESM variables during the intervention and at follow-up. In a first step, we calculated the means and standard deviations per phase. Additionally, we performed unequal variances *t*-tests and computed the Cohen’s *d* statistics (with pooled standard deviation as the denominator) to estimate effect sizes. In a second step, we fitted eight separate autoregressive lag 1 (AR (1)) models using the *lm* function in R (version 3.6.1). Dependent variables were the outcomes of interest, whereas independent variables were the lag of the dependent variables and the intervention phase, thus representing the autoregressive parameter and the mean intervention effect for each intervention phase respectively. Given that the AR (1) model assumes identical distribution of the errors throughout time, we lagged the independent variable within day and phase, resulting in a missing value for the lag at the first prompt of the day, as well as at the first prompt of a new phase. After each model, we performed a test for homoscedasticity after removing outliers, using the *outlierTest* function of the *car* package in R. In case residuals appeared to be heteroscedastic, the initial model (with outliers) was refitted using robust standard errors, by defining the robust variance-covariance matrix as argument using the function *vcov* of the *stats* package in R. For the analyses in both steps (*t*-tests, Cohen’s *d*, and AR (1) models), baseline scores were taken as the reference group. Finally, we investigated – in descriptive pie charts – how nominal ESM variables evolved throughout the intervention. All analyses were performed for each participant individually.

#### Aim 2: dynamics between motivation and related processes

To explore the dynamics of the processes associated with motivation, we used individual network representations based on vector autoregressive (VAR) modelling. We computed network models for each phase separately (baseline, intervention and follow-up). We used the *graphicalVAR* package ver. ﻿0.2.2 [38] to estimate the temporal and contemporaneous models and to obtain a visual representation using the *qgraph* package ver. 1.6.4 [39]. The nodes in the networks represent the variables, whereas the edges (i.e., the lines) represent the associations between the variables. In the temporal network, the edges are directed and indicate which variable predicts other variables in the next timepoint. In the contemporaneous network, the edges represent partial correlations between the variables, after controlling for all the other variables in the same timepoint and also in the previous timepoint. All associations reported and represented on the models are significant (*p* < .05).

We also calculated plots representing rolling means (or “moving averages”) which can be found in supplementary material, Figure S1. The course of the different processes – during baseline, intervention and at follow-up – was plotted using the *rollapply* function from the *emaph* package ver. 1.0.0 [40]. This provides rolling means, i.e., the means of each variable as it progresses over time. A rolling mean smooths the time-series, thus making it easier to detect any evolution (e.g., trend and periodicity) and to reveal any associations between variables.

## Results

Detailed descriptions of (1) the effects of Switch on motivation and related processes and (2) their dynamics are presented below for each participant individually. Results on the traditional assessment scales (BNSS, LARS, FROGS, S-QoL) are presented for each participant in Table 3.

**Table 3:** *Pre, post and follow-up scores on the traditional assessment scales and effect size coefficients for the change in the composite scores*

		Pre T0	Post T2	Follow-up T3	ES T0-T2	ES T0-T3
2i-1	BNSS-Mot^a^	1.79		1.07		
	LARS-i	−27		−32		
	LARS-p	−23		−18		
	*Motivation/Apathy*	1.35		0.99		**0.73**
	S-QoL	66.34		71.70		
	FROGS	70		86		
	*QoL/Functioning*	3.75		4.58		0.37
2i-2	BNSS-Mot^a^	3.43	2.29	3.5		
	LARS-i	−11	−15	−23		
	LARS-p	−17	−21	− 16		
	*Motivation/Apathy*	2.63	1.89	2.43	**1.40**	0.15
	S-QoL	62.93	61.46	65.37		
	FROGS	64	72	77		
	*QoL/Functioning*	3.39	3.65	3.99	**2.37**	**1.54**
2i-4	BNSS-Mot	4^a^	3.14^b^			
	LARS-p	8	−14			
	*Motivation/Apathy*	3.89	2.49		**4.34**	
	S-QoL	35.12				
	FROGS	46	60			

*Note: BNSS-Mot* Brief Negative Symptom Scale – Motivation (mean score); *LARS* Lille Apathy Rating Scale informant (i) and patient (p) (total scores); *FROGS* Functional Remission of General Schizophrenia (total score); *S-QoL* Schizophrenia Quality of Life (total score); *italics* composite variables; *bold* large effect sizes; *ES* effect size; maximum likelihood estimator, using pooled standard deviation as the denominator

^a^ BNSS Motivation average score of the two blinded evaluators

^b^ Participant 2i-4 was not filmed at T2. The BNSS Motivation was scored by one independent evaluator unaware of her score at baseline

### Participant 2i-1

#### Aim 1: treatment effects of switch on motivation and related processes

At baseline, participant 2i-1, a 29-year-old female, presented with mild motivational negative symptoms according to the BNSS, and no apathy according to the LARS. She was not assessed directly at post-measurement, potentially due to a relapse which she reported later. However, her scores at follow-up showed that motivational deficits reduced to a minimal level according to the BNSS. The score at the LARS-i also reduced. Overall, we observed a medium to large effect size coefficient for the motivation/apathy composite score at follow-up. Participant 2i-1 also reported a slightly higher quality of life. The FROGS score revealed a considerate increase in functioning. Overall, we observed a small to medium effect size coefficient for the QoL/functioning composite score at follow-up.

The effects on the ESM variables for participant 2i-1 are presented in Table 4. During the intervention phase, our analyses showed a significant increase in effort (*t* (*df*) = 3.69(101); *p* < .001; *d* = .64), and a significant effect of the intervention on this score (*b* = .82, *p* = .029). Energy (*t* (*df*) = 2.28(113); *p* = .025; *d* = .42), mood (*t* (*df*) = 4.87(97); *p* < .001; *d* = .85), confidence (*t* (*df*) = 3.44(98); *p* < .001; *d* = .60), and savouring (*t* (*df*) = 2.91(106); *p* = .004; *d* = .52) scores were significantly better. Further, there was a significant intervention effect for mood (*b* = 1.19, *p* < .001), confidence (*b* = 0.64, *p* = .008), and savouring (*b* = 0.44, *p* = .025).

**Table 4:** *Descriptive statistics regarding the different outcomes at baseline, during the intervention and at follow-up; Cohen’s d coefficients; AR (1) models b coefficients*

Participant 2i-1									
		Mean	*SD*	*t*	*df*	*d*	*CI* lower	*CI* upper	*b*
Activity’s	T0	5.13	1.64						
meaning	T1	5.53	1.24	1.66*	110	0.28	−0.04	0.60	0.32
	T3	5.06	1.46	− 0.23	112	−0.04	− 0.41	0.33	− 0.23
Motivation	T0	4.77	1.34						
	T1	4.57	1.33	−0.92	135	−0.15	− 0.47	0.17	0.24
	T3	4.84	1.17	0.30	112	0.05	−0.32	0.43	0.21
Effort	T0	4.44	2.13						
	T1	5.56	1.43	3.69****	101	0.64	0.32	0.97	0.82**
	T3	3.98	2.40	−1.07	101	−0.20	−0.58	0.17	−0.74
Energy	T0	4.48	1.53						
	T1	4.82	1.20	1.48	113	0.25	−0.07	0.57	0.39
	T3	5.08	1.26	2.28**	113	0.42	0.04	0.79	0.46
Mood	T0	5.13	1.73						
	T1	4.55	1.97	−1.95*	146	−0.31	−0.63	0.01	−0.39
	T3	6.33	0.86	4.87****	97	0.85	0.47	1.24	1.19****
Confidence	T0	5.08	1.38						
	T1	4.74	1.31	−1.56	130	−0.26	−0.58	0.07	−0.08
	T3	5.76	0.71	3.44****	98	0.60	0.22	0.98	0.64***
Savouring	T0	4.29	1.12						
	T1	4.53	1.02	1.41	127	0.23	−0.09	0.55	0.40*
	T3	4.78	0.67	2.91***	106	0.52	0.14	0.90	0.44**
Steps	T0	4949	1800						
	T1	3846	2492	−1.91*	36	−0.47	−1.06	0.11	− 616.78
	T3	3926	1699	−1.65	30	−0.58	−1.32	0.15	− 755.84
**Participant 2i-2**									
		Mean	*SD*	*t*	*df*	*d*	*CI* lower	*CI* upper	*b*
Activity’s	T0	4.50	1.70						
meaning	T1	4.30	1.72	−0.65	78	−0.12	−0.48	0.25	−0.3966
	T3	4.57	1.48	0.18	67	0.04	−0.44	0.52	−0.02
Motivation	T0	4.57	0.82						
	T1	4.32	0.74	−1.73*	71	−0.33	−0.70	0.03	−0.26*
	T3	4.30	0.64	−1.57	69	−0.36	−0.84	0.12	−0.30
Effort	T0	2.76	2.03						
	T1	3.04	1.91	0.76	73	0.14	−0.22	0.51	0.28
	T3	4.20	1.69	3.27***	68	0.76	0.26	1.25	0.76
Energy	T0	4.50	1.21						
	T1	4.22	0.94	−1.31	63	−0.27	−0.63	0.10	−0.34
	T3	4.77	0.43	1.31	54	0.27	−0.20	0.75	0.17
Mood	T0	4.00	0.91						
	T1	3.52	1.00	−2.77***	85	−0.49	−0.86	−0.12	−0.41*
	T3	3.87	1.14	−0.53	54	−0.13	−0.61	0.35	−0.10
Confidence	T0	4.31	0.90						
	T1	4.52	0.84	1.30	73	0.25	−0.12	0.61	−0.01
	T3	4.40	0.77	0.46	68	0.11	−0.37	0.58	−0.25
Savouring	T0	4.24	0.54						
	T1	3.86	0.59	−3.65****	83	−0.65	−1.03	−0.28	−0.24
	T3	4.16	0.62	−0.43	58	−0.14	−0.62	0.33	−0.06
Steps	T0	10,904	7518						
	T1	11,167	6397	0.13	21	0.04	−0.52	0.60	1129
	T3	12,959	9407	0.62	21	0.25	−0.54	1.03	3488
**Participant 2i-4**									
		Mean	*SD*	*t*	*df*	*d*	CI lower	CI upper	*b*
Activity’s	T0	3.39	1.97						
meaning	T1	4.22	1.12	2.36**	51	0.55	0.14	0.96	0.92*
	T3								
Motivation	T0	4.04	1.19						
	T1	3.81	0.91	−1.03	62	−0.23	−0.63	0.18	0.09
	T3								
Effort	T0	3.61	1.76						
	T1	4.37	1.34	2.31**	62	0.51	0.09	0.92	0.43
	T3								
Energy	T0	3.21	1.28						
	T1	3.57	1.12	1.44	70	0.30	−0.10	0.71	0.34
	T3								
Mood	T0	1.79	1.28						
	T1	1.78	0.86	−0.02	57	0.00	−0.41	0.40	0.35
	T3								
Confidence	T0	3.61	1.03						
	T1	3.48	0.95	−0.63	73	−0.13	−0.54	0.27	0.02
	T3								
Savouring	T0	2.20	1.03						
	T1	2.30	0.78	0.52	62	0.11	−0.29	0.52	0.45*
	T3								
Steps	T0	1819	1303						
	T1	4267	2800	2.71**	13	1.20	0.33	2.07	2270*
	T3								

Significance levels: * *p <* .10; ** *p* < .05; *** *p* < .01; **** *p* < .001

Notes. *SD* standard deviation; *t* t-value for difference in mean between T0-T1 and T0-T3; *df* degrees of freedom; *d* Cohen’s d; CI confidence intervals of the d coefficient; *b* AR (1) model’s b coefficient (General Least Squares); *T0* baseline; *T1* intervention; *T3* follow-up of 3 months

**Fig. 4:**
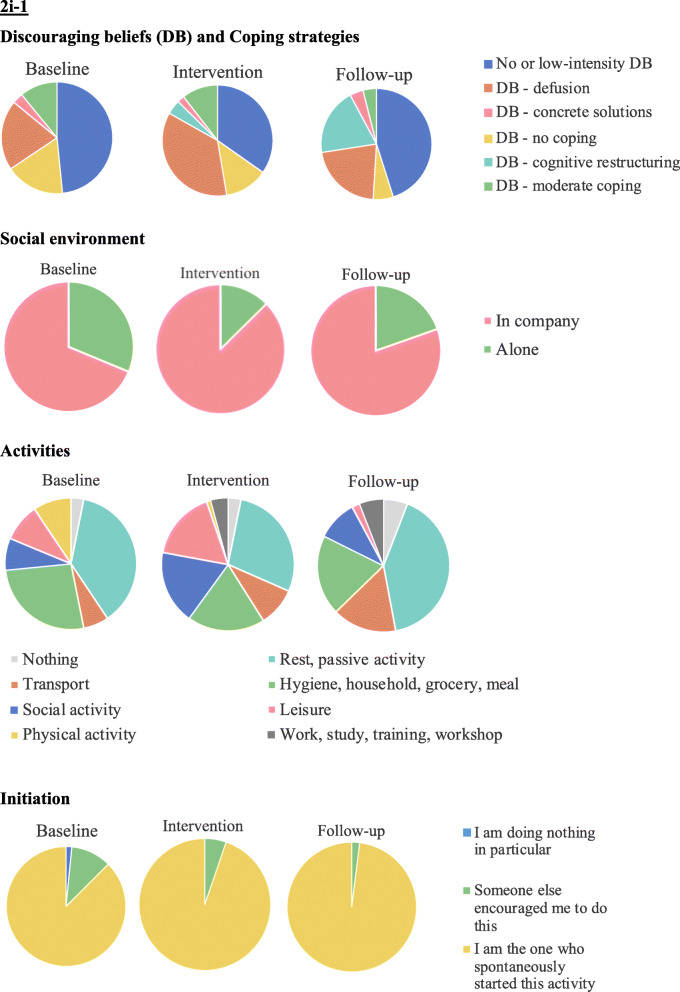
Pie charts for nominal variables of participant 2i-1

Based on the pie-charts that are presented in Fig. 4, it appears that participant 2i-1’s discouraging beliefs decreased during the intervention and at follow-up. Further, when experiencing discouraging beliefs, she appeared to cope better during the intervention and at follow-up. The occurrences of “no coping” indeed decreased. Finally, she appeared to use a more varied set of coping strategies, namely, she started using cognitive restructuring strategies during the intervention and she increased their use at follow-up. Regarding social contact, at baseline, participant 2i-1 spent most of her time in the company of other people (varying from strangers to relatives). During the intervention and at follow-up, she seemed to spend even more time in the company of other people. Regarding activities, participant 2i-1 reported that most of the time she was resting or doing a passive activity and, next in line, reported activities related to household chores. During the intervention, the proportion of passive activity decreased in favour of more social and leisure activities. This was not observed at follow-up. Finally, at baseline, participant 2i-1 was rarely not doing anything in particular and only occasionally needed someone to encourage her to engage in a certain activity. She showed even more self-initiation during the intervention and at follow-up.

#### Aim 2: dynamics between motivation and related processes

**Fig. 5:**
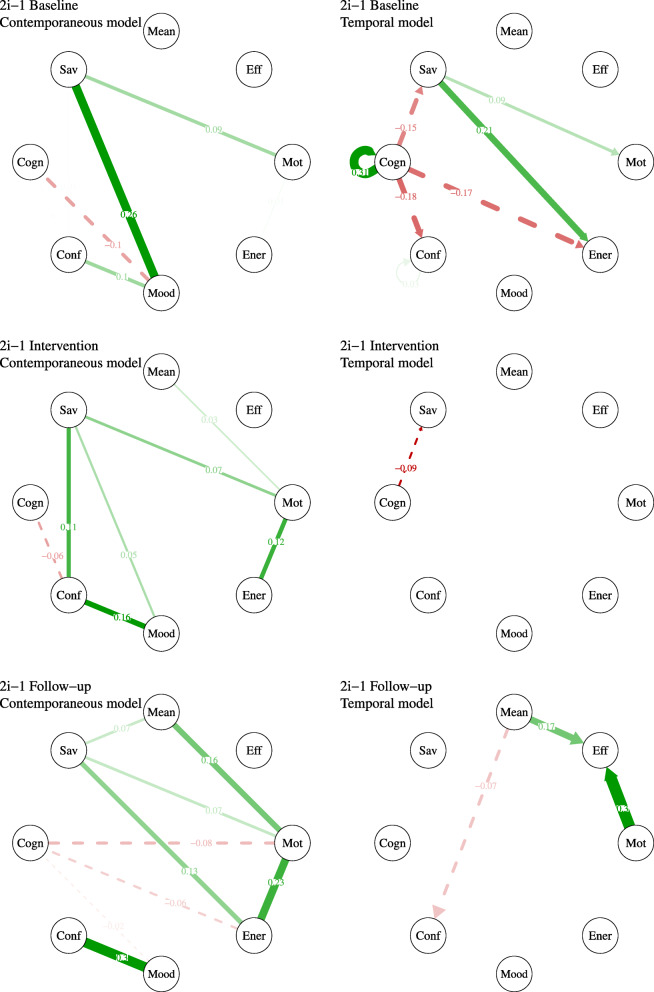
Network models of participant 2i-1. Positive associations appear in green (continuous lines) and negative associations appear in red (dashed lines). The stronger the relationships, the thicker the edges. Only associations with *p*-values < .05 are plotted. For a colour version of this figure, see the digital version of the paper

Based on the network analyses that are presented in Fig. 5, it appears that during the baseline phase, participant 2i-1’s discouraging beliefs were significantly auto-correlated (*r =* .31; see the autoregressive loop in the Baseline Temporal model). This indicates that the more she had discouraging beliefs, the more she would have discouraging beliefs also at the next time of measurement. These discouraging beliefs appeared to co-occur with lower mood (*r =* .26) (see Baseline Contemporaneous model) and appeared to impact subsequent levels of energy (*r* = *−*.17), feelings of confidence (*r = −*.18) and savouring processes (*r = −*.15) (see Baseline Temporal model). During the intervention, these associations decreased. Indeed, discouraging beliefs were no longer significantly autocorrelated, and were only slightly and negatively associated with subsequent savouring processes (*r* = −.09) (see Intervention Temporal model). This was mostly maintained at follow-up. At follow-up, motivation was shown to be significantly associated with the activity’s meaning, i.e., the more she was motivated, the more meaningful was the activity she was engaged in (*r* = .16). Furthermore, both motivation and activity’s meaning became predictive of engagement in later effortful activities (*r* = .30 and *r* = .17, respectively, see Follow-up Temporal model).

### Participant 2i-2

#### Aim 1: treatment effects of switch on motivation and related processes

At baseline, participant 2i-2, a 34-year-old male, presented with moderate to moderately severe motivational negative symptoms according to the BNSS, and moderate apathy according to the LARS. At post-measurement, motivational deficits were evaluated as being mild, and apathy scores, both LARS-i and LARS-p, decreased. Overall, we observed a very large effect size coefficient for the motivation/apathy composite score at post-measurement. There was no improvement on quality of life. The FROGS score revealed a considerate increase in functioning. Overall, we observed a very large effect size coefficient for the QoL/functioning composite score at post-measurement.

At follow-up, the improvement on the BNSS and the LARS-p was no longer observed. However, the apathy score according to the participant’s informant (LARS-i) reduced, reaching the threshold for “no apathy”. Overall, there was no significant change for the motivation/apathy composite score at follow-up. Participant 2i-2 reported higher scores on quality of life (S-QoL). The FROGS score revealed further improvement in functioning. Overall, there was a very large effect size coefficient for the motivation/apathy composite score at follow-up.

The effects on the ESM variables for participant 2i-2 are presented in Table 4. During the intervention phase, his mood significantly worsened (*t* (*df*) = − 2.77(85); *p* = .007; *d* = .86), as well as savouring (*t* (*df*) = − 3.65(83); *p* < .001; *d* = .65). There was a significant intervention effect for mood worsening (*b* = −.41; *p =* .09), but not for savouring (*b* = −.24; *p* = .12).

At follow-up, participant 2i-2 engaged in significantly more effortful activities (*t* (*df*) = 3.27(68); *p* = .002; *d* = .76). There was, however, no significant intervention effect for effort (*b* = .76; *p =* .23).

**Fig. 6:**
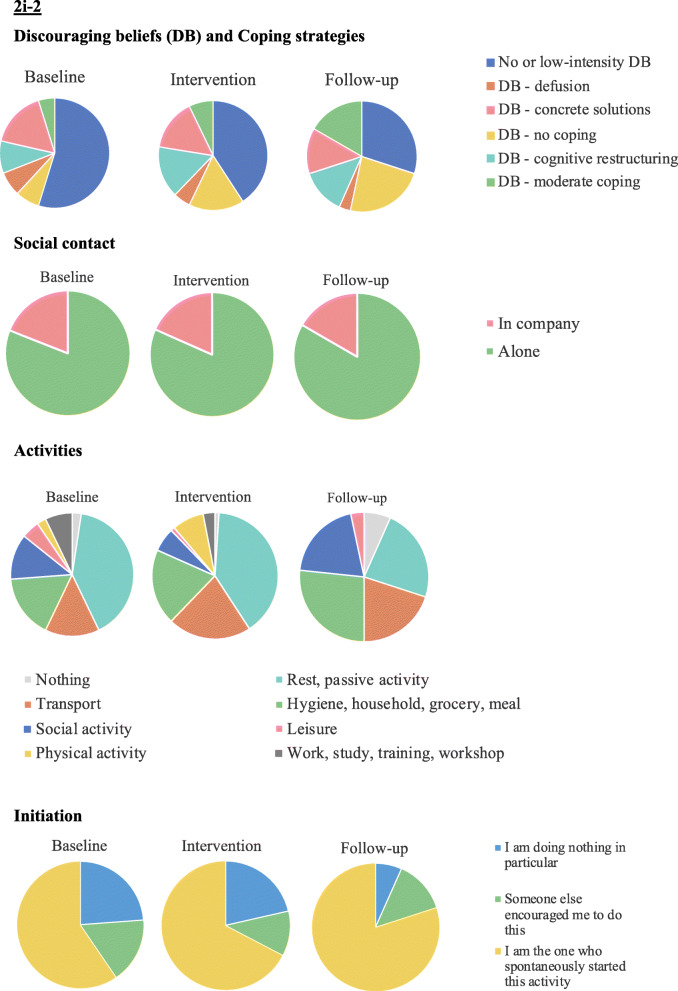
Pie charts for nominal variables of participant 2i-2

Based on the pie-charts that are presented in Fig. 6, it appears that participant 2i-2 experienced more discouraging thoughts during the course of the intervention and at follow-up. He also reported more occurrences of not coping with these thoughts. Regarding social contact, he appeared to be alone most of the time, which did not change during the intervention or at follow-up. Regarding activities, there is no apparent change from baseline to the intervention phase. At follow-up, participant 2i-2 reported less passive activity and more daily life tasks and social interactions. Finally, regarding initiation, participant 2i-2 reported less often doing nothing in particular, especially at follow-up. Moreover, he reported slightly less occurrences where someone needed to encourage him to engage in a certain activity.

#### Aim 2: dynamics between motivation and related processes

**Fig. 7:**
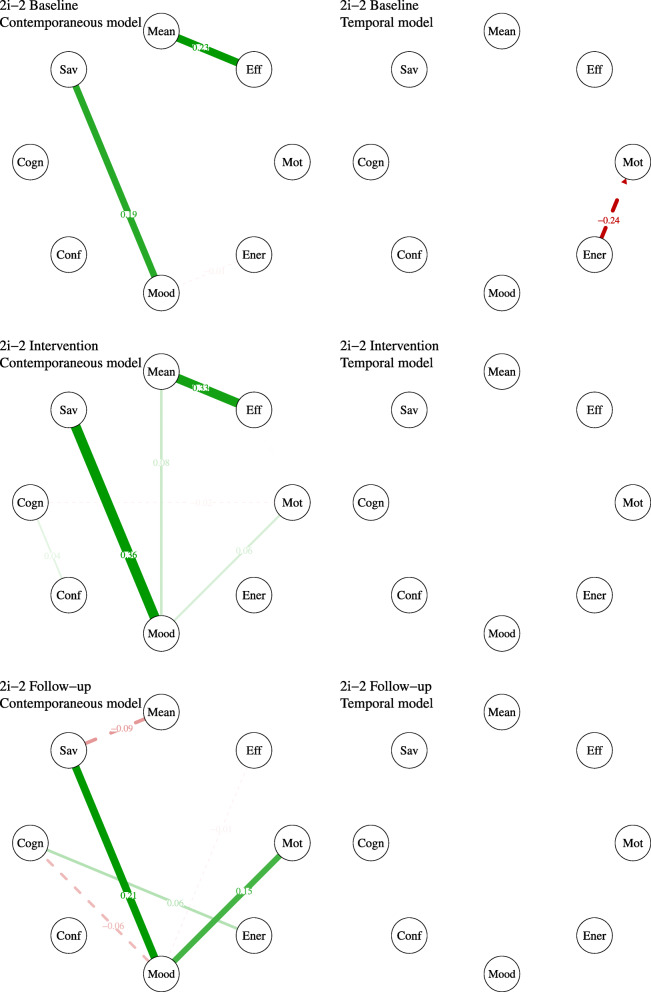
Network models of participant 2i-1. Positive associations appear in green (continuous lines) and negative associations appear in red (dashed lines). The stronger the relationships, the thicker the edges. Only associations with *p*-values < .05 are plotted. For a colour version of this figure, see the digital version of the paper

Based on the network analyses that are presented in Fig. 7, it appears that certain associations between the different variables persisted over the course of the intervention and at follow-up. Namely, savouring processes were associated with mood at baseline (*r* = .19), even more so during the intervention (*r* = .36) and at follow-up (*r* = .21). Furthermore, engaging in meaningful activities were associated with more effort at baseline (*r* = .23) and during the intervention (*r* = .33), but not at follow-up. At follow-up, motivation became significantly associated with mood (*r* = .15). The associations between the other processes were very weak.

### Participant 2i-4

#### Aim 1: treatment effects of switch on motivation and related processes

At baseline, participant 2i-4, a 39-year-old female, presented with moderately severe motivational negative symptoms according to the BNSS, and severe apathy according to the LARS-i. At post-measurement, motivational deficits were evaluated as being mild, and apathy ratings, accord to the LARS-p, reduced to moderate apathy. Everyday functioning also improved (according to the participant’s responses on the FROGS), reaching almost the threshold for remission. She did not wish to continue the assessment at follow-up.

The effects on the ESM variables for participant 2i-4 are presented in Table 4. Her ESM data reveal very low levels of mood (*M* = 1.79, *SD* = 1.28) and savouring processes (*M* = 2.20, *SD* = 1.03). During the intervention phase, she reported significantly more meaningful activities (*t* (*df*) = 2.36(51); *p* = .022; *d* = .55) and significantly more effortful activities (*t* (*df*) = 2.31(62); *p* = .024; *d* = .51). There was a significant intervention effect on meaningful activities (*b* = 92; *p* = .07). Additionally, there was a significant intervention effect on savouring processes (*b* = .52, *p* = .09). Finally, her step count increased significantly (*t* (*df*) = 2.71(13); *p* = .024; *d* = 1.20) and there was a significant intervention effect on steps (*b* = 2270; *p* = .083). Note that this was based on the data collected during baseline and the first 15 days of the intervention phase, as the step count could not be collected afterwards due to technical issues.

**Fig. 8:**
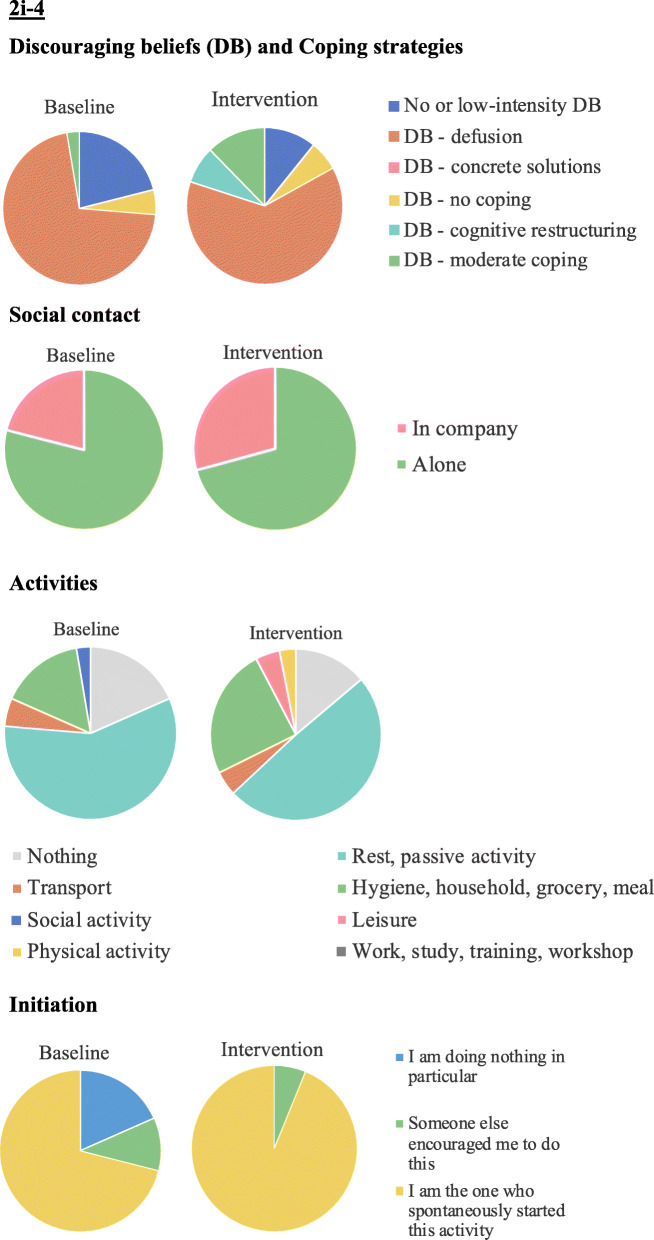
Pie charts for nominal variables of participant 2i-4

Based on the pie-charts that are presented in Fig. 8, it appears that participant 2i-4 experienced more discouraging thoughts during the course of the intervention. However, during the course of the intervention, her use of coping strategies varied and she started using cognitive restructuring (i.e., using constructive thoughts more often). Regarding social contact, she appeared to be alone most of the time, which changed slightly during the intervention, where she was less alone. Regarding activities, participant 2i-4 reported – most of the time – doing nothing, resting or doing some passive activities. During the intervention, she reported less passivity and more activities such as domestic tasks, leisure and physical activity. Finally, regarding initiation, participant 2i-4 reported more self-initiation during the intervention phase.

#### Aim 2: dynamics between motivation and related processes

**Fig. 9:**
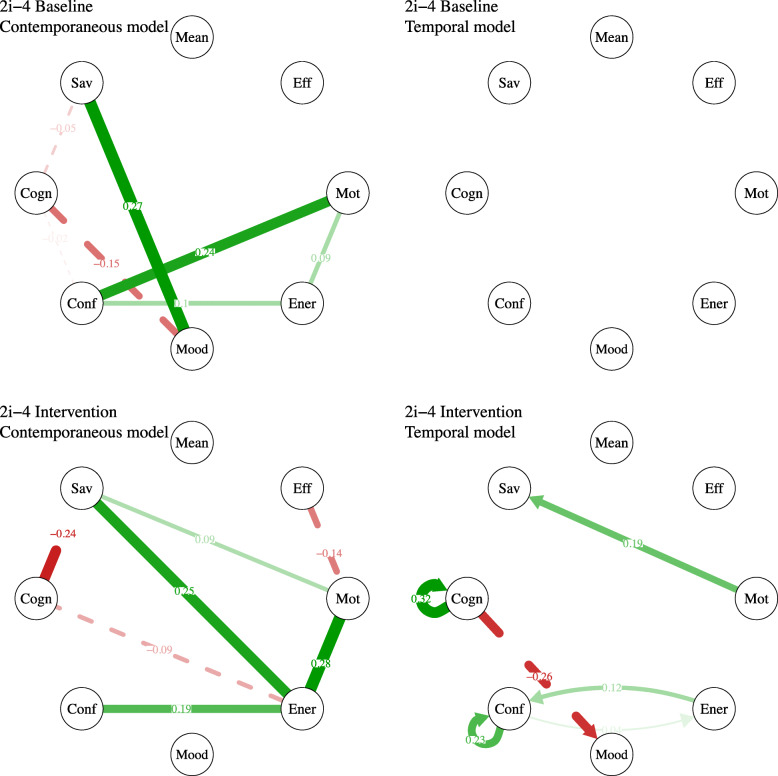
Network models of participant 2i-4. Positive associations appear in green (continuous lines) and negative associations appear in red (dashed lines). The stronger the relationships, the thicker the edges. Only associations with *p*-values < .05 are plotted. For a colour version of this figure, see the digital version of the paper

The network analyses that are presented in Fig. 9 reveal quite important changes in the dynamics of the various processes comparing baseline and intervention phases. At baseline (see Contemporaneous model), we found associations between savouring processes and mood (*r* = .27), confidence and motivation (*r* = .24), discouraging thoughts and mood (*r* = −.15), and weaker associations between confidence and energy (*r* = .10), energy and motivation (*r* = .09), and discouraging thoughts and savouring (*r* = −.05). During the intervention (see Intervention, Contemporaneous model), savouring processes became more associated with energy (*r* = .25), discouraging thoughts (*r* = −.24) and motivation (*r* = .09). This indicated that the more she used savouring skills (enjoying the present moment, looking forward to the future or reminiscing positively), the less she experienced discouraging thoughts, her energy levels increased, and she experienced more motivation – and vice versa. Furthermore, there was an increased association between energy and confidence (*r* = .19) and energy and motivation (*r* = .28), so that, the more she felt confident, the more she had energy and the more motivated she was. Moreover, the temporal influence of the different variables changed substantively (see Temporal models). At baseline, no variables predicted the other variables at the next time of measurement. During the intervention phase, motivation predicted later savouring (*r* = .19), discouraging thoughts became auto-correlated (*r* = .32) and predicted later mood (*r* = −.26), and confidence became also auto-correlated (*r* = .23) and was predicted by previous levels of energy (*r* = .12).

## Discussion

The aim of this study was to further validate Switch, an intervention that targets motivation in individuals with schizophrenia spectrum disorders. We wished to evaluate the effects of Switch on motivation, apathy, quality of life and daily functioning (primary outcomes), as well as on daily measures of various processes and outcomes related to motivation. Furthermore, we aimed to explore how the processes of interest were interrelated and developed throughout the course of the intervention, on an individual level.

The first participant we presented, participant 2i-1, appeared to have benefitted the most from the intervention, showing improvement on both traditional evaluations of motivation/apathy and functioning, as well as on processes related to motivation as measured with the ESM. However, it is not straightforward to interpret her results, as she started relapsing towards the end of the intervention, potentially because she no longer adhered to her medication in the period previous to the start of the intervention. She participated thoroughly in the intervention, attending all sessions until the 12th session. She did not come to the last 4 sessions that were planned and progressively stopped both answering the ESM questionnaires and using the activity band. However, she did learn all the strategies that Switch offered and complied with the homework, as observed during the sessions. She was present at the booster session (around 2 months after her last session). During both the booster and feedback sessions, she mentioned that the strategies she learned in the Switch intervention allowed her to hold on, which in turn could avert a “complete crisis” and subsequent hospitalisation. Furthermore, her improvement on savouring processes, confidence and coping strategies in regard of discouraging beliefs points towards a specific effect of Switch. Finally, the change in the dynamics of the different processes is interesting: during and after the intervention, discouraging thoughts lost their impact on other processes (savouring, confidence, energy) related to motivation and goal-directed behaviours.

Regarding participant 2i-2, Switch had large positive effects based on the traditional evaluations of motivation/apathy and QoL/functioning. Surprisingly, these improvements did not translate into consistent improvements on daily life measures. On the contrary, daily measures showed that he was feeling slightly less motivated, sadder and experiencing more discouraging thoughts during the intervention. However, as he explained at different times (during the intervention and at the feedback session), feeling sad was an important change for him. He felt more alive, more sensitive and reassured that he could still feel emotions despite the medical treatment. It is possible that the study procedure and the intervention made him more aware of, and sensitive to, his emotions (see for example [41, 42]). Nonetheless, we would have hoped for an increase in *positive* emotions. Regarding the follow-up evaluation, we found out after the end of the study (during the feedback session) that he stopped taking his medication before the follow-up assessment, which renders the interpretation of the follow-up results difficult. Daily measurements at follow-up revealed that he was more active and showed more self-initiation, which was not in line with the results from the traditional evaluations.

Finally, participant 2i-4 also showed improvement based on the traditional scales. Important to note is that these results rely solely on her reports, as no informant was involved. This improvement was also perceived in the ESM: she reported engaging in more activities, which appeared to be more meaningful and more effortful. Additionally, her step count increased significantly. However, these improvements were not clearly accompanied by changes on the daily measures of processes related to motivation, except for savouring, but only marginally.

Of interest, the network models of each participant reveal important differences between the participants. On the one hand, such distinctions might explain the diverse responses to the intervention. On the other hand, the network models seem to develop similarly in one way: the interconnectedness of the different variables seem to increase over the different phases of the study. Previous studies have hypothesised that a tightly connected network of symptoms was associated with higher severity [43]). In our model, we did not use symptoms, but other psychological variables including functional processes. We might thus hypothesize that an increased connection between those variables is a sign of better functioning. This would have to be further explored, comparing samples of healthy individuals with samples of individuals with different mental disorders.

Overall, while the positive impact of Switch on usual measures of motivation, apathy and daily functioning was observed in each participant, this was not consistently translated by a change in daily life measurements of motivation and activity, or processes related to motivation and steps. It is plausible that the impact on certain daily life processes (i.e., measured with the ESM) develop on a longer term [44, 45], or that the intervention itself would need to last longer in order for some cognitive or hedonic skills to improve for certain individuals. Indeed, the idiosyncratic analyses revealed very different dynamics between those processes and it appears that the intervention impacts each person differently. It is possible that the effects of Switch depend on baseline symptoms and how processes interact prior to the intervention. Further development of Switch could incorporate these individual differences by employing individual network analyses resulting from the ESM to adapt the intervention to the person’s more central processes (e.g., [46]). Taking this a step further, Switch could benefit from an Ecological Momentary Intervention approach [47], which would help individuals target the specific difficulties they meet in their daily lives by making use of a mobile intervention. Finally, weekly feedback on the ESM measures could also increase awareness of the measured processes and improve efficacy in the related skills (e.g., looking forward to the future, taking distance from discouraging thoughts) (see for example [48]).

This study has several strengths, both on a methodological and a theoretical level. First, we used different types of instruments to evaluate the effects of our intervention, combining traditional scales (completed by both participants and informants) and blind evaluations for our main outcome (BNSS Motivation), daily subjective reports via online questionnaires (ESM), and daily objective measurements via step count. Furthermore, this evaluation procedure was re-applied 3 months later at follow-up. To the best of our knowledge, this is the first study to combine an idiographic approach with ESM in order to explore processes related to motivation during an intervention for individuals with schizophrenia. Such an approach enabled an uncovering of the all-important dynamics and connectedness between variables that greatly differ from one individual to another, and this with the help of complex time series analyses. The use of ESM in clinical trials opens perspectives in the study of psychopathological phenomena, mediators of change, and potentially in the development of personalized interventions and interventions that are closely related to daily life functioning [19, 20]. Another important strength of our study is the solid theoretical foundations of the Switch intervention. Switch was specifically designed to target motivational negative symptoms, but importantly was based on a multifactorial model of motivation that encompasses various cognitive, emotional and behavioural processes.

There are certain limitations worth noting. First, the study took place in a naturalistic setting, with clear ecological advantages (e.g., generalisability of the findings to similar settings), albeit also with some drawbacks such as lack of control on certain important factors (e.g., change in medication, relapse). Second, adherence to the assessment protocol was not complete for all participants, thus limiting certain interpretations. Third, analyses did not take into consideration the varying lag that spanned between two observations, which could alter the associations we found between the variables. To date, analyses that take into account minutes or hours, rather than the prompt index (i.e., the prompt number within a day) have not been developed. Furthermore, the number of observations per participant may have been too limited to test more complex models, including for example AR (1) models with interaction effects. More intensive data would also allow the use of models able to identify mechanisms of change (e.g., vector autoregressive moving average models for multivariate prediction). Finally, the VAR analyses computed to represent the network models do not include interaction effects. Therefore, firm inferences could not be drawn regarding the effects of the intervention on the changes in the dynamical networks.

## Conclusions

Switch appears to be beneficial according to traditional measures of motivation/apathy and quality of life/daily functioning, and in some cases, regarding processes measured on a daily basis. The benefits were found in individuals with different levels (mild to severe) of severity of motivational negative symptoms and apathy. The mechanisms of change, however, could not be clearly identified. It is very plausible that the processes underlying the observed improvements vary from one individual to another. Future studies aiming to validate interventions for motivational negative symptoms should investigate the dynamics of processes related to motivation before and during interventions and, more specifically, aim to reveal these mechanisms of change. Such an approach would help the refinement of psychological interventions and guide the focus of those on strategies that target actual mechanisms of change.

## Supplementary information

**Additional file 1.**

## Data Availability

The datasets generated and analysed during the current study, as well as the intervention materials are available from the first author.

## Abbreviations

AR (1): Autoregressive lag 1 model
BNSS: Brief Negative Symptom Scale
DSM: Diagnostic and Statistical Manual of Mental Disorders
ESM: Experience Sampling Method
FROGS: Functional Remission of General Schizophrenia scale
LARS-i: Lille Apathy Rating Scale (informant version)
LARS-p: Lille Apathy Rating Scale (patient version)
S-QoL: Schizophrenia Quality of Life questionnaire
VAR: Vector Autoregressive Model
