# Supplementary material for: Idiographic analyses of motivation and related processes in participants with schizophrenia following a therapeutic intervention for negative symptoms
Source: BMC Psychiatry. 2020 Sep 25;20:464. doi: 10.1186/s12888-020-02824-5 (PMC7517640; doi:10.1186/s12888-020-02824-5)
Supplement: Supplementary file 1 — Additional file 1. [file 12888_2020_2824_MOESM1_ESM.pdf]

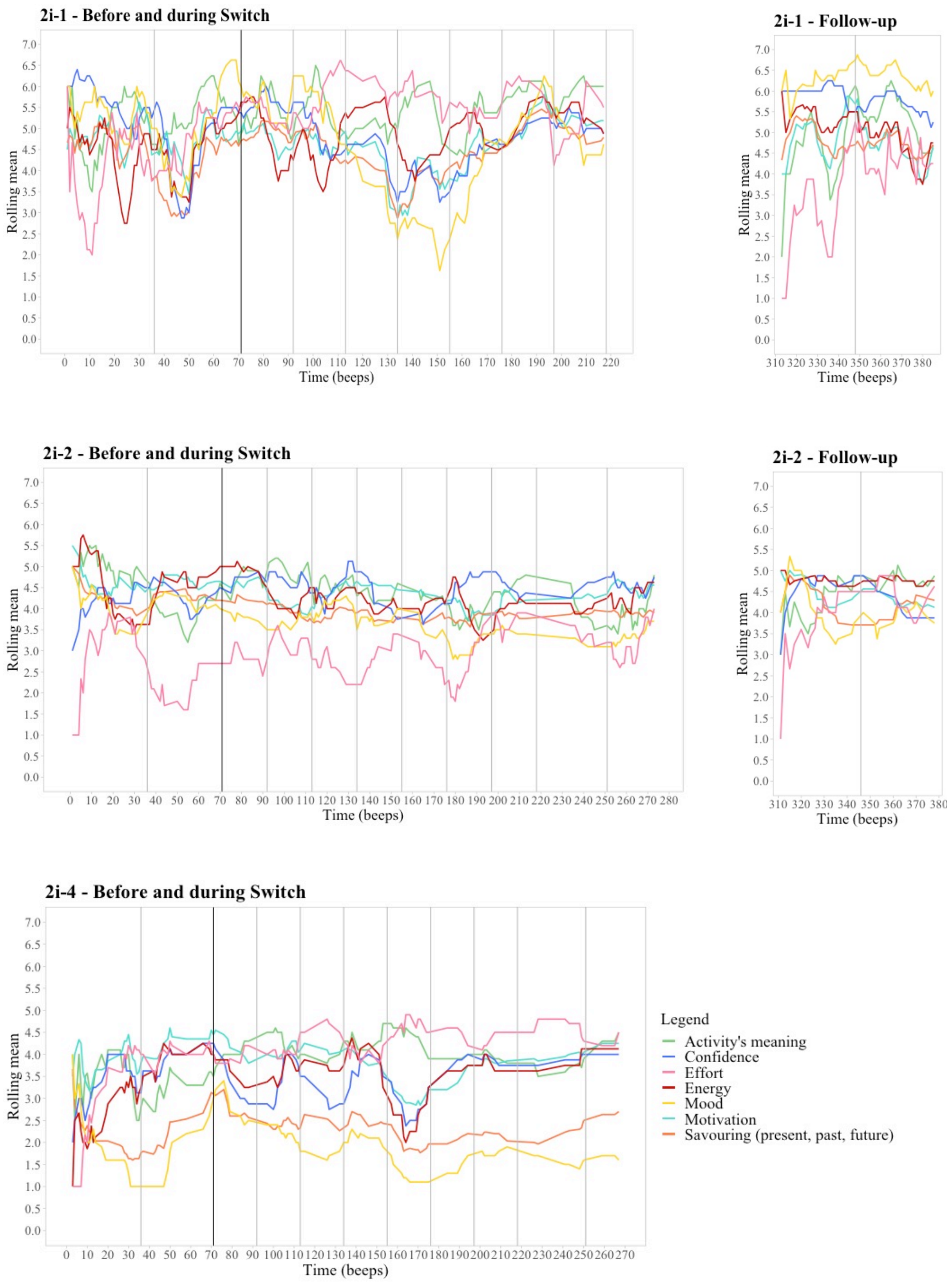

Figure S1.

Evolution of ESM variables over time for each participant, using rolling means with an 8-prompt (8-beep) window: each point on the rolling mean plot corresponds to an aggregate of 8 observations centred at that point. The vertical hard line indicates the start of the intervention. The vertical grey lines separate each week.
